# Clinical significance of true umbilical cord knot: a propensity score matching study

**DOI:** 10.1186/s12884-024-06249-w

**Published:** 2024-01-12

**Authors:** Ohad Houri, Or Bercovich, Avital Wertheimer, Anat Pardo, Alexandra Berezowsky, Eran Hadar, Alyssa Hochberg

**Affiliations:** Helen Schneider Hospital for Women, Rabin Medical Center - Beilinson Hospital, 39 Jabotinsky Street, 4941492 Petach Tikva, Israel

**Keywords:** True umbilical cord knot, Propensity score matching, Risk factors, Perinatal outcomes

## Abstract

**Objective:**

True umbilical cord knot (TUCK) is a rare finding that often leads to intensified surveillance and patient anxiety. This study sought to evaluate the incidence, risk factors, and obstetric and neonatal outcomes of TUCK.

**Methods:**

A retrospective cohort study was conducted at a tertiary university medical center in 2007–2019. Patients with singleton pregnancies diagnosed postnatally with TUCK were identified and compared to women without TUCK for obstetric and neonatal outcomes using propensity score matching (PSM).

**Results:**

TUCK was diagnosed in 780 of the 96,766 deliveries (0.8%). Women with TUCK were older than those without TUCK (32.57 vs. 31.06 years, *P* < 0.001) and had higher gravidity (3 vs. 2, *P* < 001) and a higher rate of prior stillbirth (1.76% vs. 0.43%, *P* < 0.01). Following covariate adjustment, 732 women with TUCK were compared to 7320 matched controls. TUCK was associated with emergency cesarean delivery due to non-reassuring fetal heart rate (2.54% vs. 4.35%, *P* = 0.008, OR 1.71, 95%CI 1.14–2.56) and intrapartum meconium-stained amniotic fluid (19.26% vs. 15.41%, *P* = 0.022, OR 1.31, 95%CI 1.04–1.65). Neonatal outcomes were comparable except for higher rates of 1-min Apgar score < 7 and neonatal seizures in the TUCK group. The stillbirth rate was higher in the TUCK group, but the difference was not statistically significant (1.23% vs 0.62%, *P* = 0.06, OR 1.96, 95%CI 0.96–4.03).

**Conclusions:**

TUCK has several identifiable risk factors. Pregnant women with TUCK may cautiously be informed of the relatively low risks of major obstetric or perinatal complications. The lower occurrence of stillbirth in the TUCK group warrants further study.

**Supplementary Information:**

The online version contains supplementary material available at 10.1186/s12884-024-06249-w.

## Synopsis

Using propensity score matching to avoid biases, this study showed that TUCK did not pose a drastic risk of major perinatal complications.

## Introduction

A true umbilical cord knot (TUCK) occurs when the umbilical cord twists around itself, forming a tight knot [1]. TUCK is a rare occurrence during pregnancy, with a reported incidence of 0.3 to 2% [2, 3]. TUCK formation is assumed to take place between 9 and 12 gestational weeks [4], but its detection in-utero can be challenging, and it often goes unnoticed until delivery [1, 5, 6]. Risk factors include a long umbilical cord, polyhydramnios, male fetus, gestational diabetes, and multiparity [2, 7]. Although TUCK has no clinical significance in most cases [5], blood flow to the fetus may potentially be compromised as it moves and grows within the uterus [4, 8]. This can lead to fetal hypoxia, inadequate nutrient supply, impaired waste removal, and ultimately, fetal growth restriction, fetal distress, and even stillbirth [9].

Nevertheless, the association of TUCK with intrauterine complications has not been established [8–11]. Some studies reported an increased incidence of stillbirth in association with TUCK, and findings for other adverse fetal, neonatal, and maternal outcomes have been inconsistent. With advancements in ultrasound technology and the subsequent rise in the prenatal diagnosis of TUCK, understanding its causes and implicatons has become increasingly relevant [12, 13].

The aim of this study was to evaluate risk factors and perinatal outcomes of TUCK in women diagnosed postnatally. Given that traditional multivariable analyses yielded mixed conclusions in previous outcome studies of TUCK, in the present study, propensity score matching (PSM) was used to compare variables between patients with and without TUCK in order to avoid concerns about potential confounders.

## Methods

### Study population

A retrospective cohort study was conducted in a tertiary university medical center between January 2007 and October 2019. The cohort included all women who gave birth after 22 gestational weeks or with a neonatal birthweight of at least 500g. Women with multiple gestations and terminations of pregnancy were excluded.

In our delivery ward’s protocol, all placentas are reviewed following any delivery by a midwife or an obstetrician, in order to: i) ensure that the placenta is intact and whole; 2) quantify the number of umbilical vessels; and 3) describe any umbilical vessel abnormalities, such as velamentous insertion, TUCK, etc. These findings are documented in the medical chart. TUCK was defined according to the conventional definition used in the literature (1), regardless of the degree of tightness of the knot or the number of knots.

### Data collection

Data were retrieved from the hospital’s computerized database. Women who had a postnatal diagnosis of TUCK were identified using the International Classification of Diseases, 10th Revision, (ICD-10-CM Diagnostic Code O69.2.) Maternal, neonatal, and outcome data were collected. Maternal parameters included age, gravidity, parity, history of abortions, history of a prior stillbirth, history of cesarean deliveries, use of assisted reproductive technology, body mass index (BMI), chronic hypertension, pregestational diabetes, polyhydramnios, and oligohydramnios. Neonatal parameters included gender, birthweight, 1-min and 5-min Apgar scores, umbilical artery pH, neonatal intensive care unit (NICU) admission, acidosis (umbilical cord pH < 7.2), asphyxia, seizures, and neonatal sepsis. Adverse pregnancy and delivery outcomes were defined as any hypertensive disorder of pregnancy (gestational hypertension, preeclampsia with or without severe features, or HELLP syndrome), gestational diabetes mellitus (GDM), low gestational age at delivery, mode of delivery (vaginal or cesarean delivery), the indication for cesarean delivery, meconium-stained amniotic fluid, and placental abruption.

### Outcome measures

The primary outcome was the incidence of stillbirth in the current pregnancy. The secondary outcomes were pregnancy, delivery, and neonatal outcomes in both groups. We also defined a respiratory composite outcome that included any of the following: respiratory distress syndrome, transient tachypnea of the newborn, use of continuous positive airway pressure, need for mechanical ventilation, and meconium aspiration syndrome.

### Ethics

The study was approved by the local Institutional Review Board (0132–22-RMC). Informed consent was waived due to the retrospective design.

### Statistical analysis

Categorical variables were reported as frequency and percentage. Continuous variables were assessed for normal distribution using the Shapiro–Wilk test, histograms, and Q-Q plots. Normally distributed continuous variables were reported as mean and standard deviation, and skewed variables as median and interquartile range.

PSM was used to minimize confounding. The probability of having a TUCK was calculated using a multivariable logistic regression model based on relevant patient characteristics. The model included variables previously associated with the primary outcome and those showing an association on initial univariate analysis (*P* < 0.05). Variables entered into the multivariable analysis were maternal age, gestational age at delivery, BMI category, gravidity, parity, history of abortions, history of cesarean delivery, ART, chronic hypertension, any hypertensive disorder of pregnancy, diabetes mellitus (gestational or pregestational), polyhydramnios, oligohydramnios, history of stillbirth and fetal gender. Matching was performed without replacement using a caliper of 0.001 on the propensity score scale for nearest-neighbor matching [14]. Covariate balance was assessed using standardized mean differences (SMD) before and after matching [15], with SMD < 0.1 indicating negligible differences between groups [16]. After PSM, all model variables had an SMD < 0.1. There was still a significant difference in gravidity between the matched groups, but SMD indicated a good balance. The discrimination of the propensity model was measured using the concordance (C) statistic. Overlap between patients with and without TUCK was evaluated with mirrored histograms (Figure S1). Statistical analysis was performed with unpaired methods, as the groups were dissimilar, and not based on outcome selection. Continuous variables were compared using Student's t-test or Mann–Whitney U test, and categorical variables were compared using chi-squared or Fisher's exact tests, as appropriate.

All statistical tests were two-tailed, with a significance level of *P* < 0.05/n (Bonferroni correction for number of tests). Data were generated with SPSS, version 29.0 (IBM Corp., Armonk, NY, USA) and Python, version 3.10.4.

## Results

**Fig. 1:**
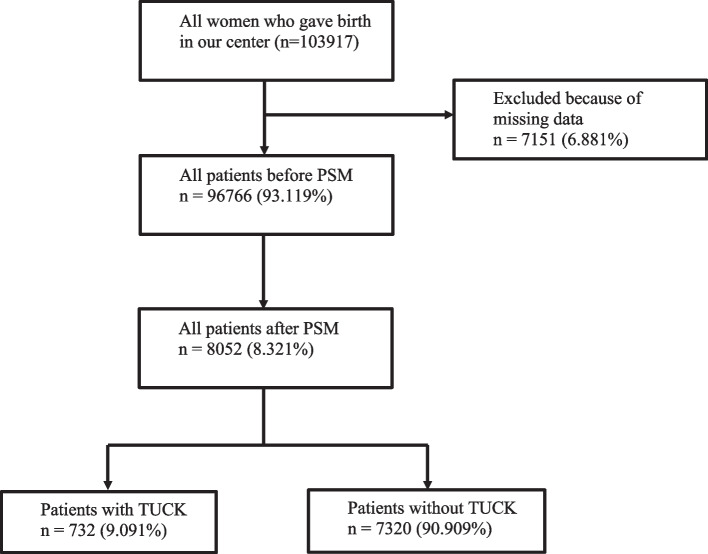
Flow diagram of patient selection. PSM, propensity score matching; TUCK, true umbilical cord knot

A total of 103,917 deliveries were performed during the study period. After excluding patients with missing data, the number was reduced to 96,766 deliveries (Fig. 1). TUCK was diagnosed in 780 deliveries, for a prevalence of 0.8%.

Maternal characteristics prior to PSM are presented in Table 1. Compared to the women without TUCK, the women with TUCK were older (median 32.56 vs. 31.05 years, *P* < 0.001), had higher median gravidity (3 vs. 2, *P* < 0.001) and parity (*P* < 0.001) and a higher rate of previous stillbirth (1.76% vs. 0.43%, *P* < 0.001), and were more likely to carry a male fetus (58.85% vs. 51.15%, *P* < 0.001).

**Table 1:** Maternal and obstetric characteristics before propensity score matching

Parameter	N	Without TUCK (*n* = 95,986)	With TUCK (*n* = 780)	*P*-value	Standardized difference	All patients (*n* = 96,766)
Maternal age,years	95,481	31.05 ± 5.28	32.569 ± 5.16	< 0.001	0.28	31.07 ± 5.29
Gestational age at delivery,weeks	95,481	39.0 [38.0–40.0]	39.0 [38.0–40.0]	0.25	-0.01	39.0 [38.0–40.0]
Gravidity	96,766	2.0 (1.0–4.0]	3.0 [2.0–4.0]	< 0.001	0.26	2.0 [1.0–4.0]
Parity	96,766	1.0 [0.0–2.0]	1.0 [1.0–2.0]	< 0.001	0.21	1.0 [0.0–2.0]
Previous abortion	96,766	0.0 [0.0–1.0]	0.0 [0.0–1.0]	< 0.001	0.19	0.0 [0.0–1.0]
Assisted reproductive technology	60,344	3309 (5.51)	23 (6.78)	0.30	0.05	3332 (5.52)
BMI < 18.5 kg/m^2^	20,878	1700 (8.24)	18 (6.84)	0.06	-0.05	1718 (8.22)
≥ 18.5 and < 25 kg/m^2^		12,611 (61.17)	145 (55.13)		-0.12	12,756 (61.09)
≥ 25 and < 30 kg/m^2^		4164 (20.19)	62 (23.57)		0.08	4226 (20.24)
≥ 30 and < 35 kg/m^2^		1489 (7.22)	24 (9.12)		0.06	1513 (7.24)
≥ 35 kg/m^2^		651 (3.15)	14 (5.32)		0.10	665 (3.18)
Previous cesarean aection	69,521	8379 (12.16)	93 (14.48)	0.07	0.06	8472 (12.18)
Hypertensive disorder of pregnancy (any)	96,722	2492 (2.59)	28 (3.80)	0.04	0.06	2520 (2.60)
Chronic hypertension	96,721	647 (0.67)	2 (0.27)	0.25	-0.05	649 (0.67)
Pre-eclampsia (any)	96,722	2194 (2.28)	16 (2.17)	0.84	-0.008	2210 (2.28)
Mild pre-eclampsia	96,721	574 (0.59)	4 (0.54)	> 0.999	-0.007	578 (0.58)
Severe pre-eclampsia	96,721	576 (0.6)	2 (0.27)	0.33	-0.05	578 (0.59)
Pre-gestational diabetes	96,722	648 (0.67)	5 (0.67)	0.82	0.0	653 (0.67)
Gestational diabetes	96,723	6232 (6.4)	56 (7.59)	0.22	0.04	6288 (6.50)
Oligohydramnion	96,722	2507 (2.61)	23 (3.12)	0.38	0.03	2530 (2.61)
Polyhydramnion	96,721	1186 (1.23)	9 (1.22)	0.97	-0.001	1195 (1.23)
Stillbirth history	96,721	418 (0.43)	13 (1.76)	< 0.001	0.12	431 (0.44)
Gender (male)	96,759	49,873 (51.15)	459 (58.85)	< 0.001	-0.15	50,332 (51.22)

Categorical variables are presented as number (%) and continuous variables as mean ± standard deviation or median (inter-quartile range)

*TUCK* true umbilical cord knot, *BMI* body mass index

Following co-variate adjustment, 732 of the 780 patients with TUCK were matched in a ratio of 1:10 with 7320 patients among the remaining cohort of patients (95,986) without TUCK (Table 2). On comparison of pregnancy and delivery outcomes, women with TUCK were at increased risk for intrapartum meconium-stained amniotic fluid (19.26% vs. 15.41%, *P* = 0.022) (Table 3). Gestational age at delivery, birthweight, and mode of delivery were not associated with TUCK. However, the incidence of emergency CD out of all women who attempted a vaginal birth (without elective CD) was also significant higher in the TUCK group (2.54% vs. 4.35%, *P* = 0.008, OR 1.71, 95%CI 1.14–2.56). Women with and without TUCK had similar rates of vacuum-assisted deliveries (21.85% vs. 21.09%, *P* = 0.314, respectively), and vacuum-assisted deliveries due to NRFHR (9.23% vs. 9.48%, *P* = 0.94).

**Table 2:** Maternal and obstetric characteristics after propensity score matching

Parameter	N	Without TUCK (*n* = 7320)	With TUCK (*n* = 732)	*P*-value	Standardized difference
Maternal age,years	8052	32.47 ± 5.36	32.52 ± 5.14	0.71	0.006
Gestational age at delivery,weeks	8052	39.0 (38.0–40.0]	39.0 (38.0–40.0]	0.40	-0.005
Gravidity	8052	3.0 [2.0–4.0]	3.0 [2.0–4.0]	0.02	0.03
Parity	8052	1.0 [0.0–2.0]	1.0 [1.0–2.0]	0.09	0.02
Previous abortion	8052	0.0 [0.0–1.0]	0.0 [0.0–1.0]	0.20	0.03
Assisted reproductive technology	8052	249 (7.44)	22 (6.52)	0.16	-0.03
BMI < 18.5 kg/m^2^	2703	161 (6.52)	18 (7.62)	0.68	0.04
≥ 18.5 and < 25 kg/m^2^		1446 (58.61)	129 (54.66)		-0.08
≥ 25 and < 30 kg/m^2^		563 (22.82)	56 (23.73)		0.02
≥ 30 and < 35 kg/m^2^		198 (8.02)	20 (8.47)		0.01
≥ 35 kg/m^2^		99 (4.01)	13 (5.5)		0.07
Previous cesarean aection	6466	767 (13.06)	86 (14.47)	0.33	0.04
Hypertensive disorder of pregnancy (any)	8052	263 (3.59)	27 (3.68)	0.89	0.005
Chronic hypertension	8052	39 (0.53)	2 (0.27)	0.58	-0.04
Pre-eclampsia (any)	8052	154 (2.10)	15 (2.05)	0.92	-0.004
Mild pre-eclampsia	8052	64 (0.87)	4 (0.54)	0.35	-0.03
Severe pre-eclampsia	8052	35 (0.47)	2 (0.27)	0.77	-0.03
Pre-gestational diabetes	8052	65 (0.88)	4 (0.54)	0.33	-0.04
Gestational diabetes	8052	538 (7.35)	54 (7.37)	0.97	0.001
Oligohydramnion	8052	243 (3.32)	22 (3.00)	0.65	-0.01
Polyhydramnion	8052	105 (1.43)	9 (1.23)	0.65	-0.01
Stillbirth history	8052	83 (1.13)	10 (1.36)	0.58	0.02
Gender (male)	8052	4311 (58.9)	434 (59.29)	0.58	-0.008

**Table 3:** Pregnancy and delivery outcomes after propensity score matching

Variables	N	Without TUCK (*n* = 7320)	With TUCK (*n* = 732)	*P*-value	OR (95%-CI)	All patients (after matching)
Normal vaginal delivery	8052	5228 (71.42)	507 (69.26)	0.31	-	5735 (71.22)
Vacuum-assisted delivery		548 (7.48)	65 (8.88)			613 (7.61)
Cesarean delivery		1544 (21.09)	160 (21.85)			1704 (21.16)
Spontaneous onset of labor	6762	3942 (64.18)	385 (62.09)	0.45	-	4327 (63.99)
Elective cesarean delivery		701 (11.41)	66 (10.64)			767 (11.34)
Induction of labor with PGE2		527 (8.58)	65 (10.48)			592 (8.75)
Induction of labor with EAB		160 (2.65)	15 (2.419)			175 (2.58)
Induction of labor with oxytocin		809 (13.17)	88 (14.19)			897 (13.26)
Emergency cesarean delivery	7351^a^	843 (12.28)	94 (13.38)	0.38	1.1 (0.88–1.38)	937 (11.63)
Emergency cesarean delivery due to NRFHR	937	168 (2.54)	29 (4.35)	0.008	1.71 (1.14–2.56)	197 (2.7)
Vacuum-assisted delivery due to NRFHR	613	52 (9.48)	6 (9.23)	0.94	0.97 (0.39–2.35)	58 (9.46)
Meconium-stained amniotic fluid	5689	797 (15.41)	100 (19.26)	0.02	1.31 (1.04–1.65)	897 (15.76)
Blood transfusions	8052	35 (0.47)	1 (0.13)	0.25	0.28 (0.04–2.08)	36 (0.44)
Intrapartum fever	8052	60 (0.82)	4 (0.54)	0.42	0.66 (0.24–1.83)	64 (0.79)
Placental abruption	8052	56 (0.76)	5 (0.68)	0.8	0.89 (0.35–2.23)	61 (0.75)

^a^After exclusion of patient who underwent elective CS

Categorical variables are presented as number (%) and continuous variables as mean ± standard deviation or median (interquartile range)

*TUCK* true umbilical cord knot, *PGE2* prostaglandin E2, *EAB* extra-amniotic balloon, *NRFHR* non-reassuring fetal heart rate

Neonatal outcomes are presented in Table 4. There were no differences between women with and without TUCK in rates of neonatal acidosis, NICU admission, infection, and composite respiratory outcome. Patients with TUCK had a higher rate of low Apgar score at 1 min (6.43% vs. 4.46%, *P* = 0.016, OR 1.47, 95% CI 1.07–2.02), as well as an increased rate of neonatal seizures (0.41% vs. 0.08%, *P* = 0.041, OR 5.01, 95% CI 1.25–20.10).

**Table 4:** Neonatal outcomes after propensity score matching

Variables	N	Without TUCK (*n* = 7320)	With TUCK (*n* = 732)	*P*-value	OR (95%-CI)	All patients (after matching)
Stillbirth	8052	46 (0.62)	9 (1.23)	0.06	1.96 (0.96–4.03)	55 (0.68)
Birthweight (grams)	8034	3200 (533.8)	3185.9 (529.1)	0.49	-	3198 (533.3)
NICU admission	7979	413 (5.68)	48 (6.66)	0.28	1.18 (0.86–1.61)	461 (5.77)
APGAR 1 min < 7	8035	326 (4.43)	47 (6.43)	0.01	1.47 (1.07–2.02)	373 (4.64)
APGAR 5 min < 7	8035	114 (1.56)	15 (2.05)	0.31	1.32 (0.76–2.27)	129 (1.60)
Fetal cord arterial PH = < 7.2	4259	174 (4.49)	18 (4.62)	0.9	1.03 (0.62–1.69)	192 (4.50)
Jaundice	8052	836 (11.42)	88 (12.02)	0.62	1.06 (0.84–1.33)	924 (11.47)
Asphyxia	8050	87 (1.18)	8 (1.09)	0.81	0.91 (0.44–1.90)	95 (1.18)
Seizure	8052	6 (0.08)	3 (0.41)	0.04	5.01 (1.25–20.10)	9 (0.11)
HIE	8052	0 (0)	0 (0)	-	-	0 (0)
IVH	8052	20 (0.27)	2 (0.23)	> 0.999	1.0 (0.23–4.28)	22 (0.27)
TTN	8052	93 (1.27)	7 (0.95)	0.46	0.75 (0.34–1.62)	100 (1.24)
RDS	8052	77 (1.05)	5 (0.68)	0.34	0.64 (0.26–1.60)	82 (1.01)
Mechanical ventialtion	8052	98 (1.33)	6 (0.820)	0.23	0.60 (0.26–1.39)	104 (1.29)
Need for CPAP	8052	24 (0.32)	1 (0.13)	0.72	0.41 (0.05–3.07)	25 (0.31)
Meconium aspiration syndrome	8052	9 (0.12)	1 (0.13)	> 0.999	1.11 (0.14–8.78)	10 (0.12)
Respiratory composite outcome^a^	8052	274 (3.74)	31 (4.23)	0.5	1.13 (0.77–1.66)	305 (3.78)
Sepsis	8052	207 (2.88)	18 (2.45)	0.56	0.86 (0.53–1.41)	225 (2.79)

Categorical variables are presented as number (%) and continuous variables as mean ± standard deviation or median (interquartile range)

*TUCK* true umbilical cord knot, *NICU* neonatal intensive care unit, *HIE* hypoxic ischemic encephalopathy, *IVH* intraventricular hemorrhage, *TTN* transient tachypnea of the newborn, *RDS* respiratory distress syndrome, *CPAP* continuous positive airway pressure

^a^Respiratory composite outcome – defined as any of the following: TTN, RDS, mechanical ventilation, meconium aspiration syndrome, use of CPAP

The rate of stillbirth was higher in the TUCK group, but the difference did not reach statistical significance (1.23% vs. 0.62%, *P* = 0.06, OR 1.96, 95% CI 0.96–4.03).

## Discussion

This study sought to identify risk factors and evaluate perinatal outcomes among women with a postnatal diagnosis of TUCK. There were five key findings: 1) The incidence of TUCK in the research cohort was 0.8%, with the TUCK group demonstrating a non-significant increase in stillbirth rate compared to those without TUCK. 2) Women with TUCK were older than those without TUCK and had higher gravidity and a history of stillbirth. 3) Using PSM, we found that TUCK was associated with an increased risk of intrapartum meconium-stained amniotic fluid and a higher rate of emergency cesarean delivery due to NRFHR. 4) Neonates in the TUCK group had a higher rate of low 1-min Apgar score and neonatal seizures than neonates in the group without TUCK. 5) All other obstetric and neonatal outcomes were similar between the two groups.

The 0.8% incidence of TUCK in this study is similar to rates reported previously, with values ranging from 0.3 to 2.1% [2, 5, 17–19]. The identified risk factors of increased maternal age, increased gravidity and parity, and history of stillbirth have been reported previously, together with higher BMI [2, 17–19], multiparity, previous spontaneous abortion, polyhydramnios, and diabetes mellitus [2, 7]. Some authors hypothesized that a portion of these factors are associated with a relatively large uterine volume, enabling vigorous fetal movements with a subsequent increase in the rate of TUCK [17, 19]. Additionally, advanced maternal age and previous spontaneous abortion are likely to be associated with multiparity [2, 7]. As in our study, TUCK tended to occur more often with male than female fetuses [2, 7].

Several previous retrospective studies examined the association of TUCK with pregnancy, delivery, and neonatal outcomes, but results were inconsistent [3, 7, 19, 20]. None examined these outcomes in a large cohort using PSM, making the present study unique and more resilient to bias.

Weissmann-Brenner et al. [19] examined perinatal outcomes (867 pregnancies with TUCK compared to 85,541 without) and found that women with TUCK were older, had a higher BMI, gravidity, and parity, and had a higher rate of induction of labor, meconium-stained amniotic fluid, and preterm delivery. Similar to our findings, in their study, pregnancies with TUCK were associated with a significantly higher rate of cesarean delivery due to NRFHR. However, unlike our findings, the overall rate of stillbirth in their study was significantly higher in the TUCK group (2.5% vs. 1%, *P* < 0.001), with pregnancies extending beyond 37 gestational weeks accounting for most of the (tenfold) difference (0.9% vs. 0.08% *P* < 0.001). Neonatal outcomes were also worse with TUCK, including hypoglycemia and need for phototherapy, with no differences compared to pregnancies without TUCK in 5-min Apgar score, NICU admissions, and number of hospitalization days [19].

Notably, although we found a higher rate of emergency cesarean delivery for NRFHR in the TUCK group compared to the non-TUCK group, the rates of vacuum-assisted deliveries for NRFHR were comparable between groups. This may possibly be due to the smaller number of cases, or due to some of these patients having an antenatally diagnosed TUCK, leading to an increased tendency for cesarean delivery which was clinician driven.

Using PSM, we found that neonates in the TUCK group had a higher rate of 1-min Apgar score < 7 and of seizures than neonates in the control group. There were no significant differences in any of the other pregnancy, delivery, and neonatal outcomes between the two groups.

Similar to our study, Linde et al. [3] (288 singleton pregnancies with TUCK vs 23,027 without) reported higher rates of low 1-min Apgar score in the TUCK group (aOR 3.93,95% CI 1.41–11.0). However, and in contrast to our findings, that study reported a fourfold higher risk of stillbirth. Joura et al. [7] (216 pregnancies with TUCK vs 21796 without) found TUCK to be associated with large-for-gestational age newborn, a longer umbilical cord, and a tenfold higher risk of stillbirth. Sørnes et al. [20] (286 pregnancies with TUCK vs 22,531 without) found a higher rate of stillbirth (1.7% vs. 0.6%, P < 0.05) and fetal acidosis (pH < 7.1) (8.33% vs. 4.03%, *P* < 0.01) in the TUCK group, but no difference compared to controls in Apgar scores and NICU admission. A possible explanation for the discrepancy between the nonsignificant increase in stillbirths in our present study (*p* = 0.06) and the significant findings in earlier ones might be our exclusion of some TUCK cases in which stillbirth may have occurred from the PSM model. Additionally, we examined perinatal outcomes in postnatally detected TUCK. We lacked data on the proportion of patients in the whole cohort who had antenatally detected TUCK, which could have altered pregnancy management and outcomes. Therefore, we believe the comparable stillbirth rates in the two groups in our cohort should be interpreted with caution.

Furthermore, although the neonatal seizure rate was increased in the TUCK group, it was still very low in both groups (3 cases in the TUCK group and 6 in the control group), limiting the robustness of this finding. Prenatal sonographic detection of TUCK has become easier and more common [21], but the management of nuchal or true knots of the umbilical cord has not been addressed to date by the International Society of Ultrasound in Obstetrics and Gynecology (ISUOG) and other expert groups [22, 23]. Some studies suggested that detection during pregnancy is important and that intensive follow-up, including induction of labor at 37 gestational weeks [20, 21, 24], may be helpful. Our results suggest that the association between TUCK and stillbirth remains uncertain, and therefore unnecessary intervention should be avoided. Continued expectant management with interval fetal testing while awaiting the onset of spontaneous labor is a viable clinical option. A prenatal sonographic diagnosis of TUCK should not serve as the sole indication for cesarean delivery.

The main strengths of our study are the large sample size, single-center setting, and uniform treatment and follow-up during pregnancy and delivery. The large sample permitted the utilization of PSM, which is a statistically robust means of using observational data to simulate a controlled experiment and isolate the predictor of interest, thereby increasing the validity of our results.

The study has several limitations. First, owing to the retrospective design and use of hospital databases, some data were missing, which could have impacted the results. These also include the percentage of pregnancies with an antepartum diagnosis of TUCK, and pregnancy follow-up in those cases versus cases with an overlooked diagnosis. Second, clinical follow-up was limited to early neonatal complications. Third, stillbirth is a rare event; therefore, a very large cohort is needed in order to determine statistically significant differences between groups. Thus, this finding of a comparable stillbirth rate between groups should be interpreted with caution, especially in a single-center setting, which may restrict generalizability to other medical practices. Finally, TUCK in our study was based on a macroscopic diagnosis made by a midwife or physician, and therefore, might have been under-reported. The degree of tightness of the knot, a hard-to-quantify parameter, and the number of knots, were not recorded or described, which could have had an unknown impact on outcomes.

In conclusion, based on a large cohort of patients and using a robust statistical design, this study suggests a comparable stillbirth rate in TUCK pregnancies as compared to pregnancies without TUCK. This finding may cautiously reassure both physicians and parturient patients and attenuate the need for intense fetal surveillance with its resultant maternal stress in cases of TUCK. Nevertheless, given the rarity of stillbirth, our findings should be interpreted with caution and be examined further in larger studies.

### Supplementary Information

**Additional file 1: Fig. S1.** Mirrored histograms showing overlap of patients with and without TUCK.

## Data Availability

The data that support the findings of this study are available on request from the corresponding author [O.H]. Restrictions apply to the availability of these data, which were used under license for this study. Data are available from the author [O.H] with the permission of the local institutional review board -Beilinson hospital.
