# Supplementary material for: Clinical significance of true umbilical cord knot: a propensity score matching study
Source: BMC Pregnancy Childbirth. 2024 Jan 12;24:59. doi: 10.1186/s12884-024-06249-w (PMC10785496; doi:10.1186/s12884-024-06249-w)
Supplement: Supplementary file 1 — Additional file 1: Fig. S1. Mirrored histograms showing overlap of patients with and without TUCK. [file 12884_2024_6249_MOESM1_ESM.docx]

**LEGEND TO SUPPLEMENTARY FIGURE**

**Fig. S1** Mirrored histograms showing overlap of patients with and without TUCK

|  | **Before matching** | **After matching** |
| --- | --- | --- |
| **Patients without TUCK** | **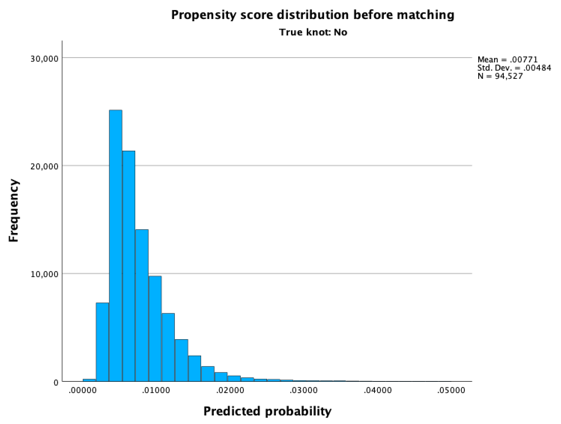** | 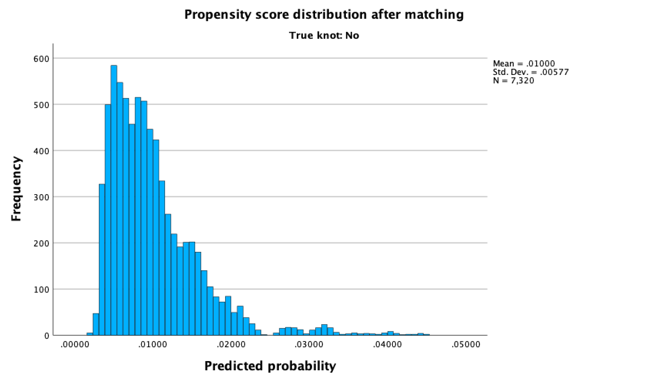 |
| **Patients with TUCK** | **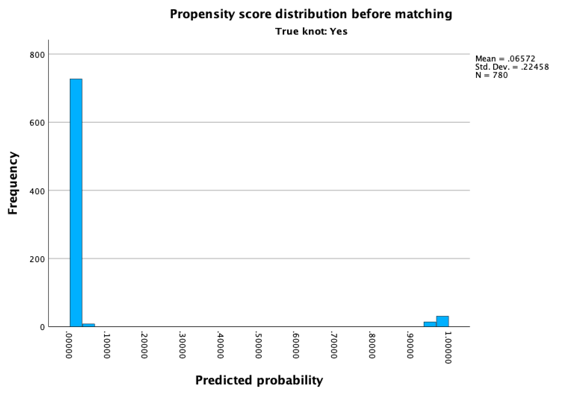** | **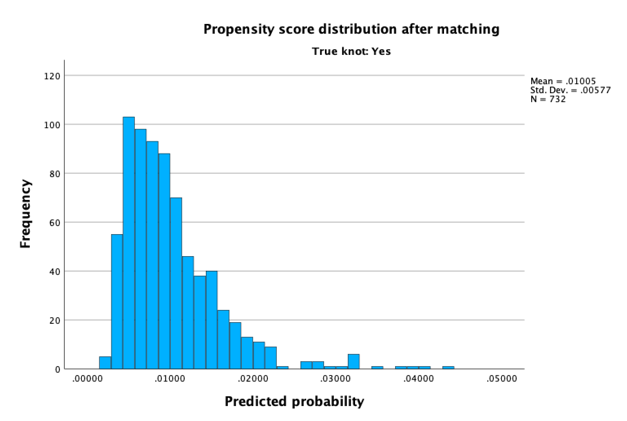** |

Abbreviations: TUCK – true umbilical cord knot
